# Comparing stress, areas of stress and coping-strategies between distance-learning and on-campus students – A mixed-methods approach

**DOI:** 10.3389/fpsyg.2022.995089

**Published:** 2022-08-18

**Authors:** Marie Drüge, Lara Fritsche, Cornelia Bögemann, Jennifer Apolinário-Hagen, Christel Salewski

**Affiliations:** ^1^Department of Psychology, Clinical Psychology and Psychotherapy Research, University of Zurich, Zurich, Switzerland; ^2^Department of Health Psychology, University of Hagen, Hagen, Germany; ^3^Faculty of Medicine, Institute for Occupational, Social and Environmental Medicine, Centre for Health and Society, Heinrich Heine University Düsseldorf, Düsseldorf, Germany

**Keywords:** coping strategies, distance-learning students, on-campus students, stress, areas of stress, mixed-methods

## Abstract

In recent years, the increase in stress experienced by students, and the related health problems have become a key challenge for health psychologists. The aim of this cross-sectional survey study was to compare stress, areas of stress and coping-strategies of 246 distance-learning (81.7% female; 33.62 years, SD = 9.30) and 254 on-campus students (82.3% female; 24.23 years, SD = 3.99). One-way analyses of variance showed no significant differences in perceived stress and stress symptoms between the student groups. Stress-inducing areas were revealed by qualitative content analysis. Chi-square tests showed that on-campus students significantly more often reported study- and performance-related areas, whereas conflicts between work and private life were more present among distance-learning students. Results also indicated that on-campus students significantly more often cope with stress by means of social support. These findings may help tailoring stress-management interventions for different student groups.

## Introduction

One focus of stress research is the subjective experience of stress and ways of coping in different target groups. In line with this, research activities on stress among students have increased since the Bologna declaration has substantially reformed and structured European academic qualification programs and more learning support and mental health strategies were called for Rückert (2015). With respect to college or university students, there is a growing body of research scoped on stress and stress-management interventions (e.g., Hintz et al., 2015; Harrer et al., 2018). Also, recently the effects of the COVID-19 pandemic on stress through online-communication and -education are evaluated and discussed (Lazarevic and Bentz, 2020; Mheidly et al., 2020) and have shown a negative psychological effect on college students around the world (Wang et al., 2021).

### Different forms of studying

During the last decades, studying has become more diverse. Most post-secondary students are so-called “traditional” students, starting university after graduating from school, while “non-traditional” students usually start their studies later in life (Jones et al., 2016)–in full- or part-time. One central distinction is made between on-campus students and distance-learning students (Furlonger and Gencic, 2014), with a growing number of distance-learning students. Students choose this form of studying mainly because of its compatibility with both work and private life (e.g., parenting). Also, the COVID-19 pandemic has led to a major shift to distance learning. However, also national differences (e.g., tuition fees) could have a large impact on differences in studying experience. It is possible that different forms of studying may result in different stressors, for example, on-campus students may experience stress due to inflexible time schedule or social interactions, whereas distance-learning students may experience stress rather due to distraction by online learning or social isolation.

### Stress, areas of stress, and coping among students

With regard to the two forms of studying, it is very likely that they are associated with different types of stressors and that the level of stress, areas of stress and coping-strategies differ between on-campus and distance-learning students, although there are only a few studies that compare the two forms of studying directly. For instance, Furlonger and Gencic (2014) reported higher levels of satisfaction in 295 on-campus students from an Australian university compared to two distance-education modes. Beccaria et al. (2015) found comparable health-promoting behaviors and coping strategies in 242 on-campus students and 399 distance students. They found a significant, though not very strong negative relationship between health-promoting behaviors and intention to leave for on-campus students.

Whereas the majority of studies on students’ stress focus on on-campus students, there are only some studies that focus on distance-learning students: Kwon et al. (2010) reported that distance-learning students were likely to experience increased feelings of social isolation. Kwaah and Essilfie (2017) found in a sample of 332 distance-learning students at a University in Ghana that the major areas of stress, in addition to academic workload and high frequency of examinations, were financial problems and family/marriage problems. These students used multiple coping-strategies, mainly meditating, self-distracting activities like watching TV and listening to music. Furthermore, in a sample of 5,721 distance-learning students of Germany’s largest distance-learning university, Apolinário-Hagen et al. (2018) confirmed a work-life-study-imbalance.

Summarizing the current state of research, there is a substantial lack of studies comparing stress, areas of stress and coping directly between on-campus and distance-learning students. Regarding the situation in Germany, there are no corresponding studies so far, but some studies aiming at stress and coping of on-campus and distance-learning students separately. Moreover, none of the studies considers both quantitative and qualitative approaches, although these prove to be helpful especially in the case of a very private experience like stress. Still, due to COVID-19, it is now more important than ever to learn more about differences in the perceived stress and areas of stress between distance-learning students and on-campus students. Although a general increase in stress caused by distance-learning was observed during the COVID-19 pandemic (Wang et al., 2021), this result needs to be interpreted within the specific context of the COVID-19 situation. For an analysis of stress-related differences between distance-learning and on-campus students that is not blurred by the exceptional situation created by the COVID-19 pandemic, we therefore present data from the time before the onset of the pandemic (i.e., 2018).

### Aims of the present study

The present study aims at comparing distance-learning students with traditional on-campus students from German universities with regard to perceived stress, areas of stress and coping-strategies.

Research Question 1: Are there differences in perceived stress and stress symptoms between distance-learning students and on-campus students?

Research Question 2: Are there differences in areas of stress among distance-learning students and on-campus students?

Research Question 3: Are there differences in the type and extent of coping-strategies between distance-learning students and on-campus students?

## Materials and methods

### Study design and procedure

We conducted a cross-sectional online survey using program (Unipark, Enterprise Feedback Suite survey, version summer 2017, Questback) in summer 2018. We used a combination of qualitative and quantitative methods (mixed-method-design), referring to a study on German on-campus students conducted by Ortenburger (2013). The overall completion time was 15–20 min. Students could take part in a raffle and win one out of ten 50€ gift vouchers.

### Sample and recruitment

A total of 500 participants were recruited by email and social media in 2018 (410 females, 89 males, 1 diverse), consisting of 254 on-campus students and 246 distance-learning students over the age of 18 years, who were matriculated at several German universities. The overall average age was 29 (SD = 8.52) years. Table 1 shows further demographic characteristics of this sample. As expected, groups differed with regard to employment status, working hours and caring for children. Ethical approval was not required for this kind of non-clinical pilot survey; the study was conducted in accordance with the Helsinki Declaration. Data was saved on a secure server of the university following German and European data security regulations.

**TABLE 1:** Sociodemographic characteristics of the study sample (*N* = 500).

	Distance-learning students	On-campus students
Sample	246	254
Age: M (SD)	33.62 (9.30)	24.23 (3.99)
**Gender**		
Male	44 (17.9%)	45 (17.7%)
Female	201 (81.7%)	209 (82.3%)
Other	1 (0.4%)	
Employment	193 (78.5%)*	146 (57.5%)*
Working hours/week: M (SD)	30.96* (12.14)	15.80* (9.38)
**Caring for children**		
Yes	83 (33.7%)*	7 (2.8%)*

*Significant differences (*p* < 0.001) using chi square tests and Analysis of Variance (ANOVA).

### Measures

Table 2 shows the measures that were used for quantitative data collection. To measure perceived stress and coping strategies, scales were used that are freely available in German (see Table 2 for further information).

**TABLE 2:** Measures used for data collection.

Construct	Scale	Author(s)	Description of scale	Response format	Consistency
Perceived stress	Perceived stress scale (PSS)	Original version: Cohen et al., 1983; German version: Herbst et al., 2016	10 items, e.g., In the last 4 weeks, how often did you feel nervous and stressed?	5-point Likert scale ranging from 0 (= disagree) to 5 (= strongly agree)	Sum-score: ≥20 high level of perceived stress, Cronbach’s α = 0.89
Stress symptoms	Subscale of the German stress and coping inventory (SCI)	Satow, 2012 ^1^	13 items, e. g., “Stress and pressure can cause physical symptoms. Which symptoms did you notice on yourself the last 6 months?”	5-point Likert scale ranging from 0 (= disagree) to 5 (= strongly agree)	Cronbach’s α = 0.85
Areas of stress			The students were asked to name three main study-related areas, which stress them since the beginning of their studies	Open question	
Coping-strategies	Subscale of the SCI	Satow, 2012 ^1^	20 items, assesses five different coping-strategies (positive thinking, active coping, social support, religion, and alcohol and cigarette consumption)	5-point Likert scale ranging from 0 (= disagree) to 5 (= strongly agree)	Cronbach’s α = 0.73

^1^Freely accessible: https://www.drsatow.de/tests/stress-und-coping-inventar/SCI-Testdokumentation.pdf.

For qualitative data collection the following open question was used: “Which three areas associated with your studies have been the most stressful since you started your studies? State at least two out of three areas.”

### Data analyses

#### Quantitative analyses

Data analysis was performed using the SPSS software package by IBM (version 25) and Microsoft Excel. Out of 717 students who gave consent, 508 respondents completed the survey (response rate: 70%). Eight outliers were deleted from the data set; non-realistic values were imputed by the mean. The Kolmogorov-Smirnov test of normality indicated violation of the assumption of normality for the stress and the stress symptoms, but not for the coping scale. Therefore, we used non-parametric tests (chi-square tests) and a one-way ANOVA to test research questions 1 and 3. Effect sizes were calculated using Cohens’ d.

#### Qualitative analysis

To test research question 2, the answers to the open question were analyzed using a qualitative content analysis with inductive category development (Mayring, 2015). Two raters independently coded all open-ended questions and derived ten subordinate categories and subcategories (see Table 3), with the resulting good interrater-reliability (kappa = 0.79). For statistical comparison between the frequencies of areas of stress between both student groups a chi-square test was used (α < 0.05).

**TABLE 3:** Superordinate categories and subcategories of areas of stress.

Subordinate category	Subcategory (numbers of mentions)			
University	• Exams, tests	(153)	Quantity of study matter, • high workload, effort of learning	(104)
	• Content of study	(82)	• Final thesis	(43)
	• Grades	(42)	• Deadlines, dates	(28)
	• Term paper	(26)	• Attendance time	(22)
	University • (organization, bureaucracy)	(18)	• Duration, standard period of study	(15)
	• Group work	(13)	• Presentations	(11)
Emotions, thoughts, personal characteristics, conditions	• Future anxiety	(31)	• Personal characteristics, emotions	(27)
	• Stress	(22)	• Motivation	(20)
	• Fear of failure	(18)	• Requirements, expectations, claims	(17)
	• Health	(10)	Doubts	(10)
Time and leisure time	• Time, time pressure	(140)	• Leisure time	(45)
Social contacts	• Private life	(59)		
Pressure and performance	Performance • (pressure), pressure to succeed	(74)	• Burden/overload/no relaxation	(17)
Occupation	• Work	(48)		
Finances	• Financial problems	(46)		
Household, everyday life, housing situation	• Housing situation	(11)	• Commuting/drive	(11)
Lack of contact, lack of aid	• Anonymity, studying alone	(15)		
Other	• other	(16)		

Only subcategories with 10 mentions or more are listed.

## Results

### Differences in perceived stress and stress symptoms

Analysis of Variance results showed no differences among stress [*F*_(1,498)_ = 2.79, *p* = 0.095, η_*p*_^2^ = 0.006] and stress symptoms [*F*_(1,498)_ = 1.96, *p* = 0.162, η_*p*_^2^ = 0.004] due to different forms of studying.

### Differences in areas of stress

Table 3 gives an overview of the reported areas of stress. For results, only superordinate categories with *n* ≥ 30 were considered. Most of the subcategories are associated with university (557) as well as emotions, thoughts, personal characteristics, conditions (155), and time, leisure (185). Areas like pressure, performance (91) and social contacts (59), were also mentioned frequently. Less people named categories like occupation (48), finances (46), household, everyday life, housing situation (22), and lack of contact, lack of aid (15). All statements which could not be aggregated to one category are listed in other (16).

According to the results of the chi-square test (see Table 4), distance-learning students reported significantly more stress regarding the subcategories *time pressure, private life* (*p* < 0.001), and *leisure time* (*p* = 0.002); on-campus students reported significantly more stress regarding categories like *exams, tests* (*p* < 0.001), *final thesis* (*p* < 0.001), and *future anxiety* (*p* = 0.007), but also in *performance (pressure)*, *pressure to succeed* (*p* = 0.034).

**TABLE 4:** Absolute (n) and relative (%) frequencies of areas of stress differentiated by on-campus students (*n* = 254) and distance-learning students (*n* = 246).

Areas of stress	On-campus students		Distance-learning students		χ^2^ (1)	*p*
				
	*n*	%	*n*	%		
Exams, test	99	39	54	22	17.06	<0.001
Time, time pressure	42	17	98	40	33.66	<0.001
Study matter^a^	48	19	56	23	1.13	0.287
Content of study	36	14	46	19	1.87	0.172
Pressure^b^	46	18	28	11	4.49	0.034
Private life	18	7	41	17	11.02	<0.001
Occupation	21	8	27	11	1.06	0.304
Financial problems	23	9	23	9	0.01	0.909
Leisure time	13	5	32	13	9.50	0.002
Final thesis	37	15	6	2	23.38	<0.001
Grades	26	10	16	7	2.26	0.133
Future anxiety	23	9	8	3	7.24	0.007

^a^High workload, effort of learning.

^b^Performance (pressure), pressure to succeed.

### Differences in coping-strategies

ANOVA results in Table 5 indicate that on-campus students use social support significantly more often as a coping-strategy than distance-learning students (*p* < 0.001), albeit with a small effect size (*d* = 0.35).

**TABLE 5:** Means and standard deviations of each SCI-coping subscale as well as results of the ANOVA for on-campus students and distance-learning students (*N* = 500).

Coping-strategies	On-campus students		Distance-learning students		*F* ^a^	*p*
				
	*M*	*SD*	*M*	*SD*		
Coping	58.2	9.40	57.20	9.16	1.44	0.230
Positive thinking	13.07	3.20	13.37	3.32	1.05	0.306
Active coping	12.74	3.23	13.25	3.37	2.97	0.086
Social support	16.44	3.38	15.13	4.03	15.45	0.001
Religion	8.70	4.34	8.49	3.99	0.33	0.569
Substance consumption^b^	7.24	3.79	6.96	3.64	0.73	0.392

^a^df = 1, 498.

^b^Alcohol and cigarette consumption.

## Discussion

To our knowledge, the present study is the first directly comparing stress, areas of stress and coping between on-campus students and distance-learning students in Germany. The study helps to compare on-campus and distance-learning students without taking up the current discussion about the influence of the online situation due to COVID-19. The COVID-19 background provides a strong bias for an actual comparison.

Research question 1 focused on perceived stress and stress symptoms. Though on-campus students rated their perceived stress levels and stress symptoms higher than distance-learning students, no significant differences were found. This finding is in line with international research by Ramos (2011), Furlonger and Gencic (2014) and Beccaria et al. (2015). Still, it is also possible that the absence of any differences occured due to the survey method (e.g., scales used) or the sample (perhaps particularly stressed or less stressed students participated). Although no significant differences were found between the perceived levels of stress, the areas of stress could still be different.

Research question 2 aimed at differences in reported areas of stress. Several differences were found between the student groups: On-campus students mention study- and performance-related areas (e.g., exams, future anxiety) more often, whereas distance-learning students report more pronounced work-privacy-conflicts (e.g., time, private life). It is important to note that distance-learning students are significantly older, more often employed, and care more often for children (Jones et al., 2016), which might moderate these results. These differences in age and life circumstances indicate different areas of stress, which underlines the need for tailored interventions based on different needs due to the form of studying (Apolinário-Hagen et al., 2018). Interestingly, some categories that could have been expected to differ between the groups were not mentioned as often (e.g., higher level of procrastination in the case of distance-learning students, lack of support).

Research question 3 focused on the type and extent of coping-strategies. In contrast to prior research indicating no differences (Ramos, 2011; Furlonger and Gencic, 2014; Beccaria et al., 2015), our data showed that on-campus students used the coping-strategy social support more often, despite a small effect size. An explanation could be that distance-learning students typically study by themselves and are less involved in the social life of universities in general. However, according to the results of research question 2, the distance-learning students did not perceive lack of support as a main area of stress. Again, age and other study-specific life circumstances might moderate these results as age and life-circumstances differ between on-campus students and distance-learning students naturalistically. Nevertheless, this result points to practical implications, like implementing platforms for knowledge sharing and support.

### Limitations

The study has a self-selective sample, which might lead to an overestimation of effect sizes due to a selection bias. In addition, the group of distance-learning students differed in sociodemographic variables. On the one hand, this limits the comparability of the two study groups; on the other hand, this represents the characteristics of the group of distance-learning students very well. Furthermore, it is important to note that the study took place pre-Corona. In addition, we do not know how many different universities the students in our sample attend, which further limits the generalizability of the data, as universities may vary in methods and technologies used. Another approach would be to look at specific technologies and their relationship to stress.

The level of stress and wellbeing of students is likely to vary over time (e.g., due to exams). Accordingly, future research should consider a longitudinal approach. Also, these data are only representative for the specific time, and do not allow wide-reaching conclusions to be drawn, for example, about post-COVID-19 differences between distance-learning and on-campus students.

In the open-question format the students had to name three areas of stress, which might have led to forced answers or leaving out important areas of stress. In addition, no assumptions about the extent of stress experienced due to the different areas can be made, which should be addressed in future studies.

The Stress and Coping Inventory (SCI) allows differentiation between six coping-strategies. Still, they cannot be categorized to problem- and emotion-focused coping, which would be interesting to analyze among students to tailor interventions.

### Practical implications

First, the findings reveal a need for stress-management interventions for students (e.g., Hintz et al., 2015) by taking different forms of studying into account. Interventions for distance-learning students should focus on time-management and methods for reducing the work-privacy conflict. Furthermore, for distance-learning students the coping-strategy social support could be an overlooked resource: Distance-universities could focus on creating more (virtual) space for social bonding and peer-to-peer-support programs (e.g., social events) or professional assistance (e.g., mentoring programs). Apparently, on-campus students seem to worry more about the future and experience more pressure to succeed. Therefore, interventions could include cognitive techniques and relaxation. Last, as distance-learning students are used to the benefit of flexibility; further research should take digital delivery formats into particular consideration (Harrer et al., 2018).

## Conclusion

Our study found comparable levels of stress among distance-learning students and on-campus students, but qualitative analysis revealed differences in the areas of stress (e.g., work vs. private life) and in the use of social support as a coping-strategy. It compares on-campus and distance-learning students without taking up the background of COVID-19. It proved to be very helpful to enrich the quantitative data with qualitative data, as this opened the opportunity to reveal differences in the areas of stress that would not have become apparent in a purely quantitative approach. Specific needs for tailored interventions taking the form of studying into account can be derived.

## Data availability statement

The raw data supporting the conclusions of this article will be made available by the authors, without undue reservation.

## Ethics statement

Ethical review and approval was not required for the study on human participants in accordance with the local legislation and institutional requirements. The study was conducted in accordance with the Helsinki Declaration. Data was saved on a secure server of the university following German and European data security regulations. Written informed consent for participation was not required for this study in accordance with the national legislation and the institutional requirements.

## Author contributions

MD, CS, and CB: conceptualization. MD, LF, and CB: methodology and writing—original draft preparation. CS: resources. CB: data curation. JA-H and CS: writing—review and editing and supervision. All authors have read and agreed to the published version of the manuscript.
